# Suspected Fetal Growth Restriction at 37 Weeks: A Comparison of Doppler and Placental Pathology

**DOI:** 10.1155/2017/3723879

**Published:** 2017-03-20

**Authors:** William M. Curtin, Karmaine A. Millington, Tochi O. Ibekwe, Serdar H. Ural

**Affiliations:** ^1^Division of Maternal-Fetal Medicine, Department of Obstetrics & Gynecology & Pathology and Laboratory Medicine, Pennsylvania State University College of Medicine, Hershey, PA, USA; ^2^Department of Pathology & Laboratory Medicine, Pennsylvania State University College of Medicine, Hershey, PA, USA; ^3^Department of Obstetrics & Gynecology, Pennsylvania State University College of Medicine, Hershey, PA, USA; ^4^Division of Maternal-Fetal Medicine, Department of Obstetrics & Gynecology & Radiology, Pennsylvania State University College of Medicine, Hershey, PA, USA

## Abstract

*Objective.* Our objective was determining if abnormal Doppler evaluation had a higher prevalence of placental pathology compared to normal Doppler in suspected fetal growth restriction (FGR) of cases delivered at 37 weeks.* Study Design.* This retrospective cohort study of suspected FGR singletons with antenatal Doppler evaluation delivered at 37 weeks had a primary outcome of the prevalence of placental pathology related to FGR. Significance was defined as *p* ≤ 0.05.* Results.* Of 100 pregnancies 46 and 54 were in the abnormal and normal Doppler cohorts, respectively. Placental pathology was more prevalent with any abnormal Doppler, 84.8% versus 55.6%, odds ratio (OR) 4.46, 95% confidence interval (CI): 1.55, 13.22, and *p* = 0.002. Abnormal middle cerebral artery (MCA) Doppler had a higher prevalence: 96.2% versus 54.8%, OR 20.7, 95% CI: 2.54, 447.1, and *p* < 0.001.* Conclusion.* Abnormal Doppler was associated with more placental pathology in comparison to normal Doppler in fetuses with suspected FGR. Abnormal MCA Doppler had the strongest association.

## 1. Introduction

Fetal growth restriction (FGR) is defined in the antenatal period as an estimated fetal weight (EFW) by ultrasound less than the 10th percentile for gestational age in the United States [1]. International consensus definition of FGR is more comprehensive and incorporates other parameters such as abdominal circumference, gestational age of onset, Doppler indices, and growth deceleration before arriving at the diagnosis of FGR [2]. Doppler evaluation of maternal, fetal, and umbilical vessels has been used in the management of suspected FGR to aid in timing of delivery and theoretically could separate the fetus with a placental problem from the constitutionally small normal fetus. Newborns that are less than the 10th percentile for gestational age are classified as small for gestational age (SGA). Methods to determine whether an SGA newborn also has pathologic growth restriction are imperfect. Obvious physical features of FGR in the SGA infant, uncommon with modern obstetric management, include decreased muscle mass and subcutaneous tissue and skin desquamation [3]. Other observations proposed for diagnosing FGR among SGA newborns include low ponderal index [4] and postnatal catch-up growth [5]. Current management in FGR is designed to avoid stillbirth, incidence 1.1–3.6%, and deliver the most mature baby as possible [6, 7]. Up to 70% of fetuses with suspected FGR may be constitutionally small normal infants and may not be at increased risk for stillbirth, and the remainder (after exclusion of birth defects, congenital infections, and chromosomal abnormalities) will have FGR presumably related to a pathologic placental process [5, 8].

The use of umbilical artery Doppler in management of suspected FGR is associated with a reduction in perinatal deaths [9]. The relationship of umbilical artery Doppler patterns in FGR to placental pathology is more straightforward when the most severe patterns, absent end diastolic velocity (AEDV) or reversed end diastolic velocity (REDV), are present [10]. In these cases, which are usually delivered markedly preterm because of nonreassuring fetal testing, there is loss of arterial vessels within the villi accounting for the abnormal Doppler patterns. In FGR at later gestational ages the villous vascular tree has a larger capacity and abnormal umbilical artery Doppler patterns are less frequent; the placental pathology is more subtle and the lesions can overlap with normal pregnancies [11]. Late-onset FGR pregnancies with uterine artery and middle cerebral artery (MCA) Doppler abnormalities have been associated with placental lesions of underperfusion [12].

We therefore chose to study the correlation of Doppler abnormalities in fetuses with suspected FGR delivered at 37 weeks' gestation at our institution in order to remove the confounding factor that gestational age has on interpretation of placental pathology and the bias toward more severe placental lesions that are seen in FGR fetuses that require preterm delivery. We hypothesized that the group of suspected FGR fetuses with abnormal Doppler would have a higher prevalence of gross and histopathologic abnormalities found in FGR as compared to the group with normal Doppler.

## 2. Methods

This was a retrospective cohort study of singleton fetuses with an ultrasound estimated fetal weight less than the 10th percentile delivered at Penn State Milton S Hershey Medical Center at 37 weeks' gestation during the time period 2011–2013. The study was approved by the Research Subjects Review Board at the Penn State Milton S Hershey Medical Center. Cohorts were divided into normal and abnormal Doppler and compared with respect to both the presence and number of gross and histopathologic findings in the placenta that were plausible in their relation to the FGR. Suspected FGR was a standard indication for submission of the placenta to pathology; thus all placentas from this group were expected to have had a pathologic examination. Pregnancies with uncertain dating, multiple gestations, fetuses with major birth defects, or viral or parasitic infections were excluded. Ascertainment of gestational age followed standard clinical and ultrasound guidelines [13].

The subjects were identified by viewing the electronic birth log for all deliveries at 37 weeks' gestation with suspected FGR. The ultrasound reports were reviewed for EFW < 10th percentile within three weeks of delivery. EFW and percentile were calculated by software using biometric parameters [14, 15]. Ultrasound measurements were performed with 2–5 MHZ curvilinear transducers using the iU22 (Philips Medical Systems, Bothell, WA). All sonography was performed by experienced sonographers dedicated to maternal-fetal medicine. EFW and head circumference to abdominal circumference ratio (HC/AC) were recorded for analysis.

Doppler measurements were obtained utilizing standard techniques [16–18]. All subjects had umbilical artery Doppler as part of their ultrasound surveillance at diagnosis of suspected FGR and with serial scans up to the time of delivery. MCA and uterine Doppler evaluation had not been utilized at all for the first year of the study period in the evaluation of suspected FGR. After the first year of the study time period, MCA Doppler was incorporated routinely into the evaluation of suspected FGR by 3 of 4 maternal-fetal medicine faculty members and not at all by one faculty member. Only 1 maternal-fetal medicine specialist also utilized uterine artery Doppler in the evaluation of suspected FGR, but only once during the pregnancy. All Doppler studies were reviewed in the GE PACS system (GE Healthcare, Chicago IL) by one maternal-fetal medicine specialist (WMC). The last measurements performed and recorded prior to delivery were used for analysis. The systolic/diastolic ratio, resistance index, pulsatility index (PI), and peak systolic velocity were calculated using the software on the machine. For the MCA Doppler the cerebroplacental pulsatility ratio (CPR) was calculated by dividing the MCA Doppler PI by the umbilical artery Doppler PI. Abnormal umbilical (PI > 95th percentile), MCA (PI or CPR < 5th percentile), or uterine artery (PI > 95th percentile) Doppler for gestational age was defined by using standard reference charts [16–18]. Subjects who had at least one abnormal Doppler of any type were placed in the abnormal Doppler cohort and subjects who had only normal Doppler were placed in the normal Doppler cohort.

Placentas were examined according to standard protocol [19]. Placental weight and gross characteristics were obtained from the placental pathology report. Placental slides were retrieved from the archive and were reviewed by a single pathologist (KAM) blinded to the Doppler categorization. Placentas that had one or more gross or histopathologic feature that could be considered contributing to FGR were classified as abnormal placentas. Those placentas with no pathologic features were classified as normal. The following gross placental features were considered abnormal: placental weight < 5th percentile for gestational age [20], single umbilical artery, marginal or velamentous cord insertion, bilobed or succenturiate placenta, and circummarginate or circumvallate placenta. Additional gross placental findings that were categorized as abnormal included infarcts, abruption, intervillous/subchorionic thrombi encompassing > 5% of placenta parenchyma, and maternal floor infarction. Histopathologic findings considered contributory to FGR included the following: increased syncytial knots, villous agglutination, increased intervillous fibrin, distal villous hypoplasia, acute atherosis, mural hypertrophy in membrane arterioles, muscularization of basal plate arteries, increased placental site giant cells in decidua basalis, immature intermediate trophoblast in decidua basalis, thin umbilical cord (diameter of the umbilical cord ≤ 8 mm), uniformly avascular villi, villous stromal-vascular karyorrhexis, villitis of unknown etiology (VUE) with obliterative fetal vasculopathy, large fetal vessel thrombosis, fetal intimal fibrin cushion, chorangiosis, nucleated red cells in capillaries, and VUE. We followed published guidelines for diagnosis for histopathologic lesions related to FGR [21–23].

Maternal demographic variables collected included age, parity, BMI, race/ethnicity, smoking history, diabetes, hypertension, and mode of delivery. Newborn information collected included gender, birthweight, ponderal index/ponderal index < 10th percentile, birthweight percentile [4], birthweight to placental weight ratio [24], NICU admission, days in hospital, hyperbilirubinemia requiring phototherapy, hypoglycemia, hypothermia, and oxygen requirement. SGA was defined as birthweight for the gestational age of 37 weeks of <2500 grams [4]. Composite neonatal morbidity was defined as at least one neonatal morbidity, including NICU admission. The data were analyzed by *t*-tests, chi-square tests, and odds ratios with 95% confidence intervals as appropriate. Statistical analysis was performed using SPSS (Chicago, IL). The primary outcome was the proportion of patients with any placental pathology. Performance characteristics of each Doppler type for identification of placental pathology were calculated. Using data by Dicke et al. [25], in a study of both preterm and term SGA infants that showed 94% with histopathologic placental lesions in the abnormal Doppler group and 64% in the normal Doppler group, a sample size of 28 patients in each cohort was calculated to show this difference with a power of 80% and significance level of 0.05.

## 3. Results

We identified 177 total patients delivered at 37 weeks' gestation for the indication of suspected FGR; 56 were excluded for EFW > 10th percentile, 8 were with fetal anomalies, and 13 were with multiple gestations, leaving a total of 100 subjects: 54, normal Doppler group and 46, abnormal Doppler group. All 100 subjects were evaluated by umbilical artery Doppler, 68 by MCA Doppler, and 39 by uterine artery Doppler. There were no umbilical artery Doppler patterns of AEDV or REDV. The mean gestational ages of the last Doppler type performed prior to delivery were 36.7 ± 0.5, 36.1 ± 1.2, and 32.9 ± 4.4 weeks for umbilical, middle cerebral, and uterine artery Doppler, respectively.

Antenatal comparisons of the cohorts abnormal versus normal Doppler are given in Table 1. Maternal age was slightly less in the abnormal group. There were no significant differences in any of the other categories. The newborn comparisons are given in Table 2. Newborns in the abnormal Doppler cohort were significantly lighter and more likely to be SGA. There were no differences in any other comparisons. Overall, 20% of newborns were admitted to the NICU and 37% experienced at least one morbidity.

The proportion of placentas with pathologic features compared by Doppler type and cohort is given in Table 3. For any Doppler type utilized, a higher proportion of placental pathology was observed if the Doppler was abnormal, OR = 4.46, 95% CI: 1.55, 13.22. Of the individual Doppler types, only an abnormal MCA Doppler was significantly associated with placental pathology compared to a normal MCA Doppler, OR = 20.7, 95% CI: 2.54, 447.1.

The performance characteristics for Doppler in the diagnosis of placental pathology are given in Table 4. Doppler had both limited sensitivity and NPV for the detection and exclusion of placental pathology, respectively. All Doppler types performed better on specificity and PPV.

Comparison of the numbers of individual placental abnormalities is given in Table 5. Infarcts were significantly more common in the abnormal Doppler group, OR = 3.87, 95% CI: 1.23, 12.67. Lesions belonging to the category of maternal vascular underperfusion [22] were more common in the abnormal Doppler cohort, OR = 3.75, 95% CI: 01.51, 9.41.

An analysis of the data comparing groups with (*n* = 69) and without placental abnormalities (*n* = 31) showed birthweights to be lower in the placental abnormality cohort, 2297.7 ± 234.7 versus 2452.3 ± 178.9 grams, *p* = 0.002. There was a higher rate of SGA newborns in the placental abnormality cohort, 42 (60.9%) versus 12 (38.7%), *p* = 0.040. There were no differences in newborn morbidities (data not shown).

To study the issue whether the MFM specialists may have been biased in selection of subjects for MCA Doppler, we analyzed cohorts for baseline characteristics and outcomes according to whether or not MCA Doppler was performed and also whether or not uterine artery Doppler was performed. These analyses are given in Tables 6, 7, 8, and 9. We found that those subjects who had MCA Doppler had no difference in their antenatal characteristics in comparison to those who did not with the exception of a lower probability of vaginal delivery, the reason for which is unclear but may be random given the number of variables analyzed. Specific ultrasound parameters, the estimated fetal weight, the proportion with EFW < third percentile, and HC/AC > 95th percentile did not differ significantly between these two groups. No differences were observed between the groups that had or did not have uterine artery Doppler. We also analyzed the data including only those subjects that had both umbilical and MCA Doppler data, *n* = 68, and found no differences in baseline characteristics and outcomes and while the overall OR of any abnormal Doppler having placental pathology increased, the results were not statistically significant from when all subjects *n* = 100 were included in the analysis. The results of these analyses are given in Tables 10, 11, and 12. Logistic regression was performed on the data in Table 10 with variables included in the model: abnormal umbilical artery Doppler, abnormal MCA Doppler, Caucasian ethnicity, maternal age, maternal BMI, EFW on ultrasound, and EFW < third percentile. An abnormal MCA Doppler was the single variable that predicted the presence of placental pathology, adjusted OR = 45.9, 95% CI: 3.46, 609.6.

## 4. Discussion

Placental pathology was significantly more common in the group of suspected FGR infants delivered at 37 weeks who had an abnormal Doppler evaluation. There was, however, a high prevalence of placental pathology even in the normal Doppler cohort. This degree of pathology in the group with normal Doppler runs counter to the assumption that the fetus with suspected FGR and normal Doppler is the constitutionally small normal fetus. Our population of FGR fetuses would mainly be considered late-onset FGR, that is, >32 weeks [26]. One partial explanation for the high prevalence of placental disease in the normal Doppler cohort would be that even uncomplicated pregnancies have some histopathologic findings. Parra-Saavedra et al. [27] showed that 78% of late-onset SGA births with normal umbilical artery Doppler had histological placental abnormalities as did 22% of AGA births. McCowan et al. [28] found that abnormal umbilical artery Doppler reflected earlier and more severe growth restriction in small for gestational age fetuses but was not independently associated with newborn morbidity. They concluded that SGA newborns with normal umbilical artery Doppler were not simply constitutionally small normal infants.

We had few abnormal umbilical Doppler patterns in our study and it is well known that in late-onset FGR umbilical artery resistance is uncommonly elevated in this group and has limited sensitivity in detecting neonatal morbidity [29]. In our study umbilical artery Doppler had the lowest sensitivity of the three Doppler types utilized for the detection of placental pathology. It is unclear why the American College of Obstetricians and Gynecologists only recommends the use of umbilical artery Doppler in the evaluation of suspected FGR [1]. Umbilical artery Doppler, in combination with MCA Doppler, detects centralization of blood flow, also known as “brain-sparing,” whereby the fetus increases the blood flow to the brain when there is hypoxia. The MCA Doppler, particularly the CPR, has been shown to have improved sensitivity over umbilical artery Doppler in detection of perinatal morbidity and mortality [29]. In addition, in fetuses with suspected FGR, there is an association between an abnormal MCA Doppler and poorer neurodevelopmental outcomes at 2 years of life [30]. An abnormal MCA Doppler in our study was strongly associated with the presence of placental pathology; these findings are in agreement with those of Parra-Saavedra et al. [12].

With respect to newborn outcomes, our study showed the abnormal Doppler group to be of lower birthweight and more likely to be classified as SGA. No difference in neonatal morbidity was noted but our study was not powered to detect differences in secondary outcomes. Overall, 32% of newborns experienced at least one morbidity and there was a 19% admission rate to the NICU. This rate of morbidity appears high and brings up questions regarding the ideal gestational age for delivery in late-onset FGR. The Disproportionate Intrauterine Growth Intervention Trial at Term showed lower neonatal intensive care unit admissions after 38 weeks in comparison to 36 to 37 weeks [31].

The strengths of our study were the uniform delivery gestational age of 37 weeks and an institutional guideline that recommends placental examination for all deliveries with suspected FGR. This allowed comparisons of placental pathology not confounded by gestational age or selection bias. The weaknesses were that this was a retrospective study, not all subjects were evaluated by uterine artery and MCA Doppler, and that the Doppler examinations did not occur at the same gestational age. The timing of the uterine artery Doppler evaluations prior to delivery with an average gestational age of 32.9 weeks, considerably shorter than the timing of the umbilical and MCA Doppler, may have been a factor in its underperformance in prediction of placental pathology.

The impact of the missing MCA Doppler data on the primary outcome variable of prevalence of placental pathology is addressed further. Had this data been present it may have strengthened the association of abnormal Doppler overall with the presence of placental pathology but could have resulted in no change or even weakened the association. We had 32 cases that did not have an MCA Doppler; of these 5 already had an abnormal umbilical artery Doppler and 1 had an abnormal uterine artery Doppler leaving 26 cases of normal umbilical artery Doppler with no MCA Doppler. From our data we know that in this group of subjects about 1/3 with a normal umbilical artery will have an abnormal MCA Doppler, so this would give an additional 9 subjects in the abnormal Doppler group. The final numbers in the abnormal Doppler group would become *n* = 55 and *n* = 45 in the normal Doppler group. Assuming all 9 subjects with an abnormal MCA Doppler would have placental pathology, a recalculation of the odds of an abnormal Doppler having placental pathology in comparison to a normal Doppler does not substantially change the results: 48/55 (87.2%) versus 30/45 (66.7%), OR = 3.43, 95% CI: 1.44, 10.63. The data from Table 12, when only the subjects with both umbilical and MCA Doppler (*n* = 68) are analyzed, appear to be in agreement with these calculations.

In suspected FGR, the presence of placental pathology could be seen as validating a placental cause for the FGR. If we considered the presence of placental pathology as representing “true” or “pathologic” FGR and the absence of placental pathology representing the constitutionally small normal fetus, our study would indicate that the latter population of fetuses (31%) actually represents a minority of suspected FGR delivered at 37 weeks. MCA Doppler had high specificity and positive predictive value for placental disease and theoretically “true” growth restriction; however it had many false negatives and consequently low negative predictive value. Ideally, one would want to exclude the constitutionally small normal fetus, so that this group could be managed with less surveillance and without mandated early term delivery; our study suggests that this separation cannot be accomplished with Doppler; thus all cases with suspected FGR would have to be managed similarly. Having an agreed-upon postnatal reference standard as to what constitutes “true” or “pathologic” fetal growth restriction may allow antenatal separation of the constitutionally small normal fetus from the truly growth restricted fetus in the future.

## 5. Conclusion

Abnormal Doppler ultrasound was significantly associated with the presence of placental pathology in this group of singleton pregnancies delivered at 37 weeks' gestation for suspected FGR. Of the three Doppler types evaluated, an abnormal MCA Doppler had the strongest association with the presence of placental pathology. This study provides further evidence and support for the use of MCA Doppler in the evaluation of suspected FGR and underscores the limitation of umbilical artery Doppler alone in FGR at later gestational ages. Further investigation and tools for separating the constitutionally small normal fetus from the FGR fetus are needed.

## Figures and Tables

**Table 1:** Abnormal versus normal Doppler antenatal comparisons in suspected FGR.

Variable^*∗*^	Abnormal Doppler	Normal Doppler	OR (95% CI)	*p* value
	*N* = 46	*N* = 54		
Maternal age	25.2 ± 5.7	27.0 ± 6.2		0.044
Maternal BMI	29.6 ± 6.6	27.4 ± 4.9		0.065
Caucasian	28 (60.9)	40 (74.1)	0.54 (0.26, 1.48)	0.158
Diabetes	4 (8.7)	3 (5.6)	1.62 (0.28, 9.78)	0.700
Hypertension	3 (6.5)	1 (1.9)	3.70 (0.34, 95.8)	0.331
Parity ≥ 1	21 (45.7)	31 (57.4)	0.62 (0.26, 1.48)	0.241
Smoking	16 (34.8)	16 (29.6)	1.27 (0.50, 3.20)	0.582
EFW ultrasound (grams)	2138.4 ± 202.0	2167.8 ± 336.8		0.606
EFW < 3rd percentile	13 (28.3)	12 (22.2)	1.38 (0.51, 3.76)	0.487
HC/AC > 95th percentile	16 (34.8)	20 (37.8)	0.91 (0.37, 2.23)	0.915
Induction of labor	38 (82.6)	43 (79.6)	1.22 (0.40, 3.99)	0.705
Vaginal delivery	33 (71.7)	41 (75.9)	0.81 (0.30, 2.16)	0.634
Cesarean for nonreassuring fetal status	5 (10.9)	1 (1.9)	6.46 (0.69, 152)	0.09
Vacuum/forceps vaginal delivery	3 (6.5)	0		0.425

OR = odds ratio, CI = confidence interval, and FGR = fetal growth restriction.

^*∗*^Results expressed in mean ± SD or number (%).

**Table 2:** Abnormal versus normal Doppler newborn comparisons in suspected FGR.

Variable^*∗*^	Abnormal Doppler	Normal Doppler	OR (95% CI)	*p* value
	*N* = 46	*N* = 54		
Birthweight (g)	2268.5 ± 246.0	2411.2 ± 193.7		0.002
SGA	32 (69.6)	22 (49.7)	3.33 (1.34, 8.34)	0.004
Ponderal index (g/cm^3^)	2.37 ± 0.33	2.43 ± 0.33		0.379
Ponderal index < 10th percentile	16 (34.8)	12 (22.2)	1.87 (0.71, 4.96)	0.163
Five-minute Apgar < 7	1 (2.17)	1 (1.9)	0.35 (0.22, 0.44)	1.000
Hospital stay (days)	3 (2–16)	3 (2–21)		0.578
NICU admission	9 (19.6)	11 (20.4)	0.95 (0.32, 2.82)	0.920
Hyperbilirubinemia phototherapy	9 (19.6)	9 (16.7)	0.95 (1.22, 3.78)	0.707
Hypoglycemia	6 (13.0)	3 (5.6)	2.55 (0.52, 13.87)	0.295
Hypothermia	5 (10.9)	7 (13.0)	0.82 (0.21, 3.18)	0.748
Oxygen requirement	4 (8.7)	5 (9.3)	0.93 (1.94, 4.37)	1.000
Composite neonatal morbidity	16 (34.8)	16 (29.6)	1.27 (0.50, 3.20)	0.582
Placental weight (g)	347.4 ± 73.4	361.6 ± 83.0		0.372
Birth/placental weight ratio	6.72 ± 1.17	6.94 ± 1.39		0.400
Umbilical cord diameter (cm)	1.20 ± 0.35	1.19 ± 0.24		0.892
Placental weight < 5th percentile	22 (47.8)	18 (33.3)	1.83 (0.6, 4.47)	0.140

OR = odds ratio, CI = confidence interval, and FGR = fetal growth restriction.

^*∗*^Results expressed in mean ± SD, number (%), or median (min–max).

**Table 3:** Prevalence of placental pathology: abnormal versus normal Doppler in *suspected FGR*.

Doppler type	Abnormal Doppler		Normal Doppler		OR (95% CI)	*p* value
	Placental pathology^*∗*^		Placental pathology			
	Yes	No	Yes	No		
Any	39 (84.8)	7 (15.2)	30 (55.6)	24 (44.4)	4.46 (1.55, 13.22)	0.002
Umbilical	12 (75)	3 (25)	57 (67.1)	28 (32.9)	1.42 (0.38, 5.81)	0.770
MCA	25 (96.2)	1 (3.8)	23 (54.8)	19 (45.2)	20.7 (2.54, 447.1)	<0.001
Uterine	16 (88.9)	2 (11.1)	13 (61.9)	8 (38.1)	4.9 (0.74, 40.90)	0.074

OR = odds ratio, CI = confidence interval, MCA = middle cerebral artery, and FGR = fetal growth restriction.

^*∗*^Results expressed in number (%).

**Table 4:** Performance of Doppler in prediction of placental pathology in suspected FGR.

Doppler	Sensitivity	Specificity	PPV	NPV
Any	55.1 (42.6, 67.1)	77.4 (58.9, 90.4)	84.5 (70.5, 93.5)	43.6 (30.3, 57.7)
Umbilical	17.4 (9.3, 28.4)	87.1 (70.2, 96.3)	75.0 (47.6, 92.7)	32.1 (22.4, 43.2)
MCA	52.1 (37.2, 66.7)	95.0 (75.1, 99.9)	96.2 (80.4, 99.9)	45.2 (29.9, 61.3)
Uterine	55.2 (35.7, 73.6)	80.0 (44.4, 97.5)	88.9 (65.3, 98.6)	38.1 (18.1, 61.6)

PPV = positive predictive value, NPV = negative predictive value, MCA = middle cerebral artery, and FGR = fetal growth restriction.

^*∗*^Results expressed in % (95% confidence interval).

**Table 5:** Occurrence of individual placental abnormality by Doppler cohort in suspected FGR.

	Abnormal Doppler	Normal Doppler
	*N* = 46	*N* = 54
Placental weight < 5th percentile	22	18
Placental configuration abnormality^*∗*^	4	8
Cord problem^*∗∗*^	12	10
Infarcts	15	6
Abruption	1	0
Intervillous thrombus > 5%	1	0
Increased pervillous fibrin	7	4
Subchorionic thrombus excessive	2	1
Increased syncytial knots	1	3
Villous agglutination	3	2
Distal villous hypoplasia	0	1
Decidual atherosis	2	0
Hypertrophy membrane arterioles	0	1
Muscularization of basal plate arteries	2	0
Avascular terminal villi	0	1
Large villus intimal fibrin cushion	0	1
Chorangiosis	1	1
VUE	3	0

FGR = fetal growth restriction.

*∗* includes succenturiate lobe, circummarginate, or circumvallate membrane insertion.

*∗∗* includes thin cord, marginal or velamentous insertion, and single umbilical artery.

**Table 6:** MCA Doppler versus no MCA Doppler antenatal comparisons in suspected FGR.

Variable^*∗*^	MCA, yes	MCA, no	OR (95% CI)	*p* value
	*N* = 68	*N* = 32		
Maternal age	25.9 ± 6.1	27.9 ± 5.8		0.114
Maternal BMI	28.9 ± 6.3	27.2 ± 4.5		0.119
Caucasian	46 (67.6)	22 (68.8)	0.95 (0.35, 2.56)	0.912
Diabetes	5 (7.4)	2 (6.3)	1.19 (0.19, 9.47)	1.000
Hypertension	3 (4.4)	1 (3.1)	1.43 (0.12, 37.2)	1.000
Parity ≥ 1	36 (52.9)	16 (50.9)	1.13 (0.46, 2.83)	0.784
Smoking	25 (36.8)	7 (21.9)	2.08 (0.72, 6.18)	0.138
EFW ultrasound (grams)	2164.2 ± 196.5	2133.2 ± 412.0		0.689
EFW < 3rd percentile	18 (23.5)	9 (28.1)	1.27 (0.44, 3.64)	0.621
HC/AC > 95th percentile	23 (33.8)	13 (40.6)	1.34 (0.52, 3.46)	0.509
Induction of labor	54 (79.4)	27 (84.4)	1.40 (0.41, 5.01)	0.555
Vaginal delivery	42 (61.8)	27 (84.4)	3.34 (1.05, 11.35)	0.023

OR = odds ratio, CI = confidence interval, MCA = middle cerebral artery, and FGR = fetal growth restriction.

^*∗*^Results expressed in mean ± SD or number (%).

**Table 7:** MCA Doppler versus no MCA Doppler newborn comparisons in suspected FGR.

Variable^*∗*^	MCA, yes	MCA, no	OR (95% CI)	*p* value
	*N* = 68	*N* = 32		
Birthweight (g)	2340.6 ± 241.6	2356.3 ± 205.0		0.752
SGA	39 (57.4)	15 (46.9)	1.52 (0.60, 3.87)	0.327
Ponderal index (g/cm^3^)	2.40 ± 0.33	2.41 ± 0.33		0.862
Ponderal index < 10th percentile	20 (29.4)	8 (25.0)	1.25 (0.44, 3.63)	0.647
Five-minute Apgar < 7	0 (0)	1 (3.1)		0.320
Hospital stay (days)	3 (2–16)	3 (2–21)		0.505
NICU admission	15 (22.1)	5 (15.6)	1.53 (0.45, 5.43)	0.453
Hyperbilirubinemia phototherapy	13 (19.1)	5 (27.2)	1.28 (0.37, 4.61)	0.672
Hypoglycemia	6 (8.8)	3 (9.4)	0.94 (0.19, 5.13)	1.000
Hypothermia	8 (11.8)	4 (12.5)	0.82 (0.21, 3.18)	1.000
Oxygen requirement	6 (8.8)	3 (9.4)	0.93 (0.23, 4.07)	1.000
Composite neonatal morbidity	21 (30.9)	11 (34.4)	0.85 (0.32, 2.29)	0.727
Placental weight (g)	363.1 ± 79.7	337.9 ± 74.6		0.135
Birth/placental weight ratio	6.64 ± 1.15	7.26 ± 1.50		0.024
Umbilical cord diameter (cm)	1.20 ± 0.30	1.20 ± 0.30		1.000
Placental weight < 5th percentile	26 (38.2)	14 (43.8)	0.80 (0.31, 2.04)	0.600

OR = odds ratio, CI = confidence interval, MCA = middle cerebral artery, and FGR = fetal growth restriction.

^*∗*^Results expressed in mean ± SD, number (%), or median (min–max).

**Table 8:** Uterine artery Doppler versus no uterine artery Doppler antenatal comparisons in suspected FGR.

Variable^*∗*^	Uterine artery	Uterine artery	OR (95% CI)	*p* value
	Yes	No		
	*N* = 39	*N* = 61		
Maternal age	25.2 ± 5.8	27.4 ± 6.1		0.079
Maternal BMI	28.5 ± 5.6	28.3 ± 5.0		0.844
Caucasian	22 (43.6)	22 (75.5)	0.42 (0.16, 1.09)	0.052
Diabetes	4 (10.3)	3 (4.9)	2.21 (0.39, 13.4)	0.427
Hypertension	2 (5.1)	2 (3.3)	1.60 (0.15, 16.8)	0.642
Parity ≥ 1	24 (61.5)	28 (45.9)	1.89 (0.77, 4.65)	0.127
Smoking	16 (41.0)	16 (26.2)	1.96 (0.76, 5.04)	0.122
EFW ultrasound (grams)	2122.0 ± 203	2174 ± 322		0.362
EFW < 3rd percentile	11 (28.2)	14 (23.0)	1.32 (0.48, 3.62)	0.554
HC/AC > 95th percentile	11 (28.2)	25 (41.0)	0.57 (0.22, 1.46)	0.194
Induction of labor	30 (76.9)	51 (83.6)	0.65 (0.21, 2.0)	0.406
Vaginal delivery	24 (61.5)	45 (73.8)	0.57 (0.22, 1.47)	0.197

OR = odds ratio, CI = confidence interval, and FGR = fetal growth restriction.

^*∗*^Results expressed in mean ± SD or number (%).

**Table 9:** Uterine artery Doppler versus no uterine artery Doppler newborn comparisons in suspected FGR.

Variable^*∗*^	Uterine artery	Uterine artery	OR (95% CI)	*p* value
	Yes	No		
	*N* = 39	*N* = 61		
Birthweight (g)	2338.4 ± 263.0	2350.3 ± 207.6		0.804
SGA	39 (57.4)	15 (46.9)	1.52 (0.60, 3.87)	0.327
Ponderal index (g/cm^3^)	2.42 ± 0.35	2.39 ± 0.31		0.658
Ponderal index < 10th percentile	12 (30.8)	16 (26.2)	1.25 (0.47, 3.32)	0.622
Five-minute Apgar < 7	0 (0)	1 (3.1)		0.320
Hospital stay (days)	3 (2–16)	3 (2–21)		0.496
NICU admission	9 (23.1)	11 (18.0)	1.36 (0.45, 4.08)	0.539
Hyperbilirubinemia phototherapy	5 (12.8)	13 (21.3)	0.54 (0.15, 1.85)	0.281
Hypoglycemia	3 (3.5)	6 (9.8)	0.76 (0.14, 3.76)	1.000
Hypothermia	3 (11.8)	9 (14.8)	0.48 (0.10, 2.14)	0.358
Oxygen requirement	5 (12.8)	4 (6.6)	2.10 (0.45, 10.14)	0.306
Composite neonatal morbidity	14 (35.9)	21 (34.4)	1.07 (0.42, 2.69)	0.880
Placental weight (g)	355.4 ± 73.8	354.8 ± 82.1		0.972
Birth/placental weight ratio	6.75 ± 1.19	6.89 ± 1.37		0.594
Umbilical cord diameter (cm)	1.24 ± 0.32	1.18 ± 0.28		0.309
Placental weight < 5th percentile	17 (43.6)	23 (37.7)	1.23 (0.52, 3.14)	0.558

OR = odds ratio, CI = confidence interval, and FGR = fetal growth restriction.

^*∗*^Results expressed in mean ± SD, number (%), or median (min–max).

**Table 10:** Abnormal versus normal Doppler antenatal comparisons in suspected FGR data for *n* = 68 subjects with both umbilical artery and MCA Doppler evaluation.

Variable^*∗*^	Abnormal Doppler	Normal Doppler	OR (95% CI)	*p* value
	*N* = 41	*N* = 27		
Maternal age	25.1.2 ± 5.8	27.0 ± 6.4		0.065
Maternal BMI	29.8 ± 6.8	27.6 ± 5.2		0.065
Caucasian	24 (58.5)	22 (81.5)	0.32 (0.09, 1.14)	0.065
Diabetes	4 (9.8)	1 (3.7)	2.81 (0.27, 70.0)	0.641
Hypertension	3 (7.3)	0		0.271
Parity ≥ 1	19 (46.3)	17 (63)	0.51 (0.17, 1.53)	0.179
Smoking	15 (36.6)	10 (37.0)	0.98 (0.32, 3.03)	0.970
EFW ultrasound (grams)	2120.3 ± 198.9	2230.7 ± 176.1		0.022
EFW < 3rd percentile	13 (31.7)	3 (11.1)	3.71 (0.84, 18.76)	0.079
HC/AC > 95th percentile	15 (36.6)	8 (29.6)	1.37 (0.43, 4.43)	0.553
Induction of labor	34 (82.9)	20 (74.1)	1.70 (0.45, 6.78)	0.337
Vaginal delivery	33 (71.7)	41 (75.9)	0.81 (0.30, 2.16)	0.634
Cesarean for nonreassuring fetal status	5 (12.9)	0		0.144
Vacuum/forceps vaginal delivery	1 (3.6)	0		1.00

OR = odds ratio, CI = confidence interval, and FGR = fetal growth restriction.

^*∗*^Results expressed in mean ± SD or number (%).

**Table 11:** Abnormal versus normal Doppler newborn comparisons in suspected FGR data for *n* = 68 subjects with both umbilical artery and MCA Doppler evaluation.

Variable^*∗*^	Abnormal Doppler	Normal Doppler	OR (95% CI)	*p* value
	*N* = 41	*N* = 27		
Birthweight (g)	2269.0 ± 253.3	2449.2 ± 177.0		0.019
SGA	14 (34.1)	6 (27)	3.13 (1.02, 9.83)	0.025
Ponderal index (g/cm^3^)	2.37 ± 0.33	2.43 ± 0.33		0.379
Ponderal index < 10th percentile	14 (34.1)	6 (22.2)	1.82 (0.53, 6,4)	0.291
Five-minute Apgar < 7	0	0		
Hospital stay (days)	3 (2–16)	3 (2–12)		0.570
NICU admission	9 (22.0)	6 (22.2)	0.98 (0.27, 3.71)	0.979
Hyperbilirubinemia phototherapy	9 (22.0)	4 (14.8)	1.62 (0.39, 7.2)	0.464
Hypoglycemia	6 (14.6)	0		0.074
Hypothermia	5 (12.2)	3 (11.1)	0.82 (0.20, 6.58)	1.000
Oxygen requirement	4 (9.8)	2 (7.4)	1.35 (0.19, 11.63)	1.000
Composite neonatal morbidity	16 (39)	5 (18.5)	2.82 (0.79, 10.58)	0.073
Placental weight (g)	353.0 ± 74.4	378.4 ± 88.2		0.210
Birth/placental weight ratio	6.60 ± 1.09	6.70 ± 1.26		0.737
Umbilical cord diameter (cm)	1.20 ± 0.35	1.19 ± 0.21		0.867
Placental weight < 5th percentile	19 (46.3)	7 (25.9)	2.47 (0.77, 8.17)	0.090

OR = odds ratio, CI = confidence interval, and FGR = fetal growth restriction.

^*∗*^Results expressed in mean ± SD, number (%), or median (min–max).

**Table 12:** Prevalence of placental pathology: abnormal versus normal Doppler in suspected FGR for *n* = 68 subjects with umbilical and MCA Doppler evaluations.

Doppler type	Abnormal Doppler		Normal Doppler		OR (95% CI)	*p* value
	Placental pathology^*∗*^		Placental pathology			
	Yes	No	Yes	No		
Any	35 (85.4)	6 (14.6)	13 (48.1)	14 (51.9)	6.28 (1.75, 23.5)	0.001
Umbilical	9 (75.0)	3 (25.0)	39 (69.6)	17 (30.4)	1.31 (0.27, 7.02)	1.000
MCA	25 (96.2)	1 (3.8)	23 (54.8)	19 (45.2)	20.7 (2.54, 447.1)	<0.001
Uterine	15 (88.2)	2 (11.8)	13 (61.9)	8 (38.1)	4.62 (0.69, 38.6)	0.136

OR = odds ratio, CI = confidence interval, MCA = middle cerebral artery, and FGR = fetal growth restriction

^*∗*^Results expressed in number (%).
